# Serum Uric Acid and Renal Transplantation Outcomes: At Least 3-Year Post-transplant Retrospective Multivariate Analysis

**DOI:** 10.1371/journal.pone.0133834

**Published:** 2015-07-24

**Authors:** Kun Zhang, Baoshan Gao, Yuantao Wang, Gang Wang, Weigang Wang, Yaxiang Zhu, Liyu Yao, Yiming Gu, Mo Chen, Honglan Zhou, Yaowen Fu

**Affiliations:** Department of Urology/Transplant Center, First Hospital of Jilin University, Changchun, Jilin, China; University of Toledo, UNITED STATES

## Abstract

Since the association of serum uric acid and kidney transplant graft outcome remains disputable, we sought to evaluate the predictive value of uric acid level for graft survival/function and the factors could affect uric acid as time varies. A consecutive cohort of five hundred and seventy three recipients transplanted during January 2008 to December 2011 were recruited. Data and laboratory values of our interest were collected at 1, 3, 6, 12, 24 and 36 months post-transplant for analysis. Cox proportional hazard model, and multiple regression equation were built to adjust for the possible confounding variables and meet our goals as appropriate. The current cohort study lasts for 41.86 ± 15.49 months. Uric acid level is proven to be negatively associated with eGFR at different time point after adjustment for age, body mass index and male gender (standardized β ranges from -0.15 to -0.30 with all P<0.001).Males with low eGFR but high level of TG were on CSA, diuretics and RAS inhibitors and experienced at least one episode of acute rejection and diabetic issue were associated with a higher mean uric acid level. Hyperuricemia was significantly an independent predictor of pure graft failure (hazard ratio=4.01, 95% CI: 1.25-12.91, P=0.02) after adjustment. But it was no longer an independent risk factor for graft loss after adjustment. Interestingly, higher triglyceride level can make incidence of graft loss (hazard ratio=1.442, for each unit increase millimoles per liter 95% CI: 1.008-2.061, P=0.045) and death (hazard ratio=1.717, 95% CI: 1.105-2.665, P=0.016) more likely. The results of our study suggest that post-transplant elevated serum uric acid level is an independent predictor of long-term graft survival and graft function. Together with the high TG level impact on poor outcomes, further investigations for therapeutic effect are needed.

## Introduction

For post-transplant recipients, the outcomes and mortality of kidney were the most critical problems. Unlike short-term outcome, the long-term graft/patient survival has not significantly been improved by advanced immunosuppressant. Therefore endeavors to develop effective means that could improve long-term outcomes directly or indirectly are needed [1]

Chronic allograft nephropathy (CAN), also known as sclerosing allograft nephropathy, is the leading cause of kidney transplant failure[2] and happens months to years after the transplant. It is characterized by interstitial fibrosis, tubular atrophy, fibrotic intimal thickening of arteries and glomerulosclerosis. Death with functioning graft is another common causes of graft loss after transplantation, in which, the leading cause of death with functioning graft is cardiovascular event(CV)[3, 4]. Given this situation, one can postulate that a management attempt of either could be beneficial for long-term outcome. Theoretically, both of these outcomes share similar pathophysiological procedures such as hypertension, dyslipidemia, and insulin resistance[5]. And an increasing number of evidence showed us serum uric acid (UA) level may probably associate with these pathological processes.

At cellular and molecular level, uric acid and hyperuricemia play a role in progression of CV event and renal disease. UA induces endothelial cell dysfunction[6–9] and decreased nitric oxide production[9, 10]; it stimulates vascular smooth muscle cell proliferation and inflammatory factors[10, 11], and promotes T-cell activation through macrophage/monocyte stimulation[12]. UA has been associated with the genesis of hypertension[13] by up-regulating renin-angiotensin system[14]. Also, inflammatory markers, including C-reactive protein, interleukin-6, and tumor necrosis factor-α, are correlated with UA levels according to some reports[15, 16].

In epidemiological studies, independent associations between hyperuricemia and myocardial infarction, ischemic stroke CV events and CV mortality are solid[17–21]. Predictive value of increased UA level was obviously reflected in ESRD and kidney disease incidence[22–26]. Additionally, reduction of UA level by using allopurinol could delay the progression of hypertension and renal disease [27, 28].

In experimental models, mild hyperuricemia causes glomerular hypertension and blood pressure-independent small vessel disease in the kidney and promotes progression of renal disease in remnant kidney model[14, 29–31]. Random control trial in cyclosporine-treated rats[32] indicated that hyperuricemia leads to arteriolar hyalinosis, tubular injury and intersititial fibrosis.

Practically, the prevalence of hyperuricemia in transplant recipients is relatively common [33]. Enough proof has been acquired, allowing us to make hypothesize that an adverse effect of elevated UA level on renal transplant long-term outcomes could be possible. If this theory turns out to be valid, aggressive measures to control UA level would play a proactive role in improving graft survival/function. Plus, investigations on this phase are of limit. Therefore, it is proposed that we should evaluate the association between UA level and graft function and survival post-transplant.

## Materials and Methods

### Study design

This is a retrospective cohort study of consecutive renal allograft recipients transplanted at the Urology/Transplant center of the First Hospital of Jilin University between January 2008 and December 2011. Before surgery, Patients had undergone an extensive medical evaluation for patients’ general status, comorbidity and corresponding immunocompatibility tests. Specifically, recipients with a negative lymphocytotoxicity test result, PRA<10%, and at least one HLA match are of suitability for transplantation in our center. Donor sources were either from living-related donors or DCD (donation after cardiac death) donors involved in Urology/Transplant center of the First Hospital of Jilin University.

The study period (2008–2011) was chosen to provide a reasonable sample size and an at-least 3 years follow-up data after transplantation. All recipients were eligible for this analysis, in order to maintain consistency and continuity of this cohort. Corresponding serum creatinine (SCr) levels were used to estimated glomerular filtration rate (eGFR) during the same period with a new equation [34, 35] fit better in Chinese population. Patients were divided into hyperuricemia and normal UA groups. Hyperuricemia was defined as mean serum UA (calculated from UA values accessed at multiple times post-transplant in order to assure UA exposure) level more than 7.0 mg/dl for men and more than6.0 mg/dl for women.

### Ethic statements

The current study is specially approved by the first hospital of Jilin University ethics committee board. All activities involved are consistent with the Principles of the Declaration of Istanbul as outlined in the “Declaration of Istanbul on Organ Trafficking and Transplant Tourism” and the principles expressed in the Declaration of Helsinki. Transplant surgeon would signed the consent form (see S1 File) with living-related (immediate relative or spouse) donor together with their counterpart recipient before surgery carried out. Family of potential DCD donors would be informed by the Red Cross Society of China that they may think about organ donation based on current situation. Intention-to-donation family would fill in the *Red Cross Society of China Human Organ Donation Volunteers Registration Form* (see S2 File) so we could proceed the transplantation (organ quality assessment process would take place simultaneously). In rare cases, people had already registered as a volunteer for organ donation before unexpected accident occurred. None of the transplant donors were from a vulnerable population and all donors or next of kin provided written informed consent that was freely given.

### Immunosuppression protocol

Given their economic conditions, the major part of the recipients did not receive immunosuppression induction as our institutional protocol recommended the rest part received either anti-CD25 antibody or thymoglobulin (ATG). Basiliximab (Simulect, Norvartis Pharmaceuticals, NJ) was administrated 20 mg on the operation day and post-surgery day 4, and Daclizumab (Zenapax, Roche Pharmaceuticals, SH), which was dosed 1mg/kg on the operation will be secondly dosed on post-operative day 14. ATG (Genzyme, Cambridge, MA) was given at 2.5 mg/kg/day for 5–7 days starting intraoperatively. All recipients received methylprednisolone 500 mg IV per day intraoperatively, followed by the same dose on the next two subsequent days after surgery, with tapering to oral prednisone at 120 mg on post-transplant day 3 when IV methylprednisolone was done. And the oral prednisone would be reduced 20 mg/d until the current dosage was 20 mg. Prednisone was further tapered, achieving 10 mg/d by 6 months. Mycophenolate mofetil (CellCept, Roche, Nutley, NJ) was initiated on the next day of surgery by patients’ body weight (BW<50 kg, 500 mg twice daily; 50 kg<BW<70 kg, 750 mg twice daily; BW>70 kg, 1000 mg twice daily), and dose adjustments were made for gastrointestinal intolerance and bone marrow suppression. Tacrolimus (TAC) or Cyclosporin (CSA) was added to the regimen when the SCr was at or less than 4.0 mg/dl (initiate dosage: 0.15 mg/kg daily for TAC; 8 mg/kg daily for CSA) and adjusted by monitoring its plasma concentration and patients’ renal/hepatic function.

### Follow-up

Patients were followed up according to the recommended guidelines which indicated that all organ receivers should be returning back at the follow-up house for blood routine test; hepatic/renal function; metabolic status and immunosuppression serum concentration/AUC (area under curve). Once suspicious symptoms and signs or unexplained increase in SCr and proteinuria during follow-up period was investigated, further allograft ultrasound, Lung CT scan and graft biopsy would be made to confirm acute rejection (AR) or infection. Biopsies were evaluated using Banff ‘97 criteria for evidence of acute rejection. Patients with delayed graft function (DGF) defined as the need for dialysis in the first week post-transplant.

### Outcomes

The primary outcome of this analysis was the attempt to testify the predictive value of UA level and the possible factors that might influence UA level. The secondary outcome of our study was graft survival and its correlation with hyperuricemia and UA level. Graft loss was defined as graft failure (return to dialysis) or death with functioning graft. Pure graft failure and death with functioning kidney were considered to be split outcomes of graft loss for further analysis.

### Statistical Analysis

Data are presented as mean ± SD or n (%), as appropriate. The groups were compared using Student’s t test, chi-square or Fisher’s exact test, when needed. Survival analysis methods including Kaplan-Meier and Cox Proportional Hazard Model were used to evaluate the independent association of UA level measured at multiple time points with graft survival after adjustment for other time-dependent and independent potential covariates. To examine the independent association between UA level and eGFR, linear regression modeling was used with adjustment for potential confounding variables, including the corresponding mean eGFR. Also, logistic regression was used to model the odds ratios (OR) for hyperuricemia. Regression model assumptions were tested with proper statistical diagnostics. To check for collinearity, variance inflation factors were calculated for the predictor variables. SPSS 19.0 (Inc., Chicago, IL) and GraphPad Prism (GraphPad Software 5.0, San Diego, CA) were used for statistical analysis and figure layout.

## Results

### Baseline characteristics

There were 573 kidney transplant recipients followed up for 41.86 ± 15.49 months are included in this cohort study. Overall, 155 patients (27.1%) were hyperuricemic and the rest 418 patients (73.0%) had normal UA level. Demographic characteristics and laboratory findings of the recruited patients are summarized in Table 1. Basically, the general data of the two groups were largely comparable.

**Table 1:** Demographic characteristics and laboratory findings of the recruited patients. BMI: Body mass index; HTN: Hypertension; GN: Glomerulonephritis; AAN: Aristolochic acid nephropathy; Basi: Basiliximab; Dacl: Daclizumab; DGF: delayed graft function SCr: Serum creatinine TC: Total cholesterol; TG: Triglycerides;

Variables	Study cohort	Hyperuricemia	Normal Uric acid	
Mean ± SD or n (%)	N = 573	N = 155 (27.05)	N = 418 (72.95)	P
Age(y)	41.37 ± 9.45	41.52 ± 8.99	41.33 ± 9.61	.864
Female/Male	181(31.59)/392(68.41)	61(39.35)/94(60.65)	120(28.71)/298(71.29)	.015
BMI (kg/m^2^)	21.91 ± 2.62	22.33 ± 2.63	21.78 ± 2.61	.077
Follow-up time(m)	41.86 ± 15.49	39.91 ± 16.60	42.53 ± 15.10	.155
Donor (deceased/living)	465(81.15)/108(18.85)	129(83.23)/26(16.77)	336(80.38)/82(19.62)	.473
HLA mismatch	4.27 ± 0.87	4.34 ± 0.84	4.25 ± 0.89	.437
Pregnancy (Female %)	144(79.56)	47(77.05)	97(80.83)	.563
Original disease (GN/AAN/Others)	447(78.01)/100(17.45)/26(4.54)	122(78.71)/25(16.13)/8(5.16)	325(77.75)/75(17.94)/18(4.31)	.815
Comorbid HTN/DM/Gout	*223(38*.*92)*/25(4.36)/34(5.93)	56(36.13)/4(2.58)/11(7.10)	167(39.95)/21(5.02)/23(5.50)	.359
Dialysis(Hemo-/ Peritoneal)	530(92.50)/43(7.50)	137(88.39)/18(11.61)	393(94.02)/25(5.98)	.031
Dialysis time(m)	19.96 ± 12.92	21.82 ± 13.91	19.31 ± 12.56	.180
Hot/cold Ischemia time(min)	7.74 ± 2.08/261 ± 93.6	7.75 ± 1.89/267 ± 85.8	7.73 ± 2.16/258.6 ± 96.6	.954/.443
Induction (None/ Basi/ATG/ Dacl)	459(80.10)/73(12.74)/13(2.27)/28(4.89)	122(78.71)/17(10.97)/6(3.87)/10(6.45)	337(80.62)/56(13.40)/7(1.67)/18(4.31)	.257
Immuno suppression (CSA/FK506/Others)	150(26.18)/289(50.44)/134(23.38)	59(38.06)/65(41.94)/31(20.00)	91(21.77)/224(53.59)/103(24.64)	.000
DGF	13(2.27%)	8(5.16)	5(1.20)	.009
SCr (mg/dL)	1.10 ± 0.29	1.21 ± 0.34	0.94 ± 0.24	.000
eGFR(mL/min/1.73m^2^)	92.29 ± 18.81	81.74 ± 17.28	95.51 ± 19.13	.000
Uric acid(mg/dL)	5.87 ± 1.35	6.94 ± 1.47	5.22 ± 1.04	.000
TC(mmol/L)	5.17 ± 0.89	5.24 ± 0.84	5.19 ± 0.92	.745
TG(mmol/L)	1.88 ± 0.71	1.96 ± 0.72	1.88 ± 0.69	.032
Globulin(g/L)	25.03 ± 3.28	24.84 ± 3.17	24.96 ± 3.10	.115
Infection	85(14.83)	32(20.65)	53(12.68)	.024
Rejection	51(8.90)	20(12.90)	31(7.41)	.048
Death	26(4.54)	7(4.51)	19(4.54)	1.00
Graft loss	30(5.24)	18(11.61)	12(2.87)	.002

### Donors’ information

We included 333 donors in total, 240 (72.1%) DCD donors and 93 (27.9%) living-related donors separately. The most common cause of DCD donor’s death is car accident (196, 81.7%). Subsequent causes of death are cerebrovascular accident (21, 8.8%), cardiovascular accident (15, 6.3%) and brain tumor (8, 3.3%). The donors conformed to the organ quality certification in general. See Table 2 for more detailed information.

**Table 2:** General information of recruited donors. Abbreviations as in Table 1. Two types of donors we recruited are basically comparable in renal function, age and BMI.

Donor type	Total	DCD donor	Living donor	P value
Case number	333	240	93	
Age (y)	45.56 ± 8.22	41.23 ± 9.54	55.32 ± 4.12	0.023
Female/Male	102(30.6%)/231(69.3%)	39(16.3%)/201(83.7%)	63(67.7%)/30(32.3%)	0.000
BMI (kg/m^2^)	21.43 ± 2.43	21.12± 2.54	22.32 ± 2.21	0.451
SCr (mg/dL)	0.84 ± 0.13	0.83 ± 0.12	0.86 ± 0.15	0.565
eGFR(mL/min/1.73m^2^)	103.4 ± 20.9	109.3 ± 23.21	98.4 ± 16.98	0.184

### Basal and undergoing values of UA and eGFR with according pharmacological regimens

Time-varying values UA, Hyperurecimia percentage, eGFR, Cyclosporine plasma concentrations (C2, known as the “peak value”), tacrolimus plasma concentrations, MMF and prednisone doses, use of RAS (renin—angiotensin System) inhibitor and diuretics at different time points post-transplant are summarized in Table 3. UA levels had a slow upward trend, increasing from 5.72 ± 1.37 mg/dL at 1 month to 6.36 ± 1.42 mg/dL at 3years, together with small increase in eGFR (from85.7 ± 27.1 to 96.0 ± 26.6)during the same period. There was a steady downward trend in the immunosuppressive agent levels/doses during the first 3 years. Prescribed RAS inhibitor was lifting since the 1 month post-transplant, along with the same trend in terms of diuretics use.

**Table 3:** Post-transplantvalues of time-dependent variables. Abbreviations as in Table 1. Cyclosporine plasma concentrations are C2 values, also known as the “peak value”, which is highly associated with AUC0-4.

Variables	1 month	3 month	6 month	1 year	2 year	3 year
UA (mg/dL)	5.72 ± 1.37	6.03 ± 1.42	6.20 ± 1.67	6.31 ± 1.40	6.40 ± 1.39	6.36 ± 1.42
Hyperurecimia (%)	16.2	24.1	28.6	30.9	36.0	42.8
eGFR(mL/min/1.73m^2^)	85.7 ± 27.1	91.3 ± 25.0	92.9 ± 21.5	94.3 ± 23.9	94.9 ± 25.4	96.0 ± 26.6
Cyclosporine (ng/mL)	1552.2 ± 413.5	998.6 ± 369.0	924.8 ± 360.1	814.9 ± 367.8	761.2 ± 254.7	702.8 ± 214.1
Tacrolimus (ng/mL)	10.1 ± 5.3	8.4 ± 2.8	7.2 ± 2.3	6.4 ± 2.7	5.2 ± 2.4	5.0 ± 2.1
MMF (mg/day)	1542 ± 524	1408 ± 644	1224 ± 736	1098 ± 756	856 ± 458	834 ± 320
Prednisone (mg/day)	20.4 ± 12.2	16.6 ± 5.2	10.4 ± 4.8	8.4 ± 3.5	6.3 ± 2.1	5.8 ± 1.5
RAS inhibitor (%)	10.5	16.8	22.3	29.5	35.7	36.9
Diuretics (%)	11.4	12.7	13.8	15.3	15.6	16.2

### Predictive value of UA and its predictor

**Fig 1:**
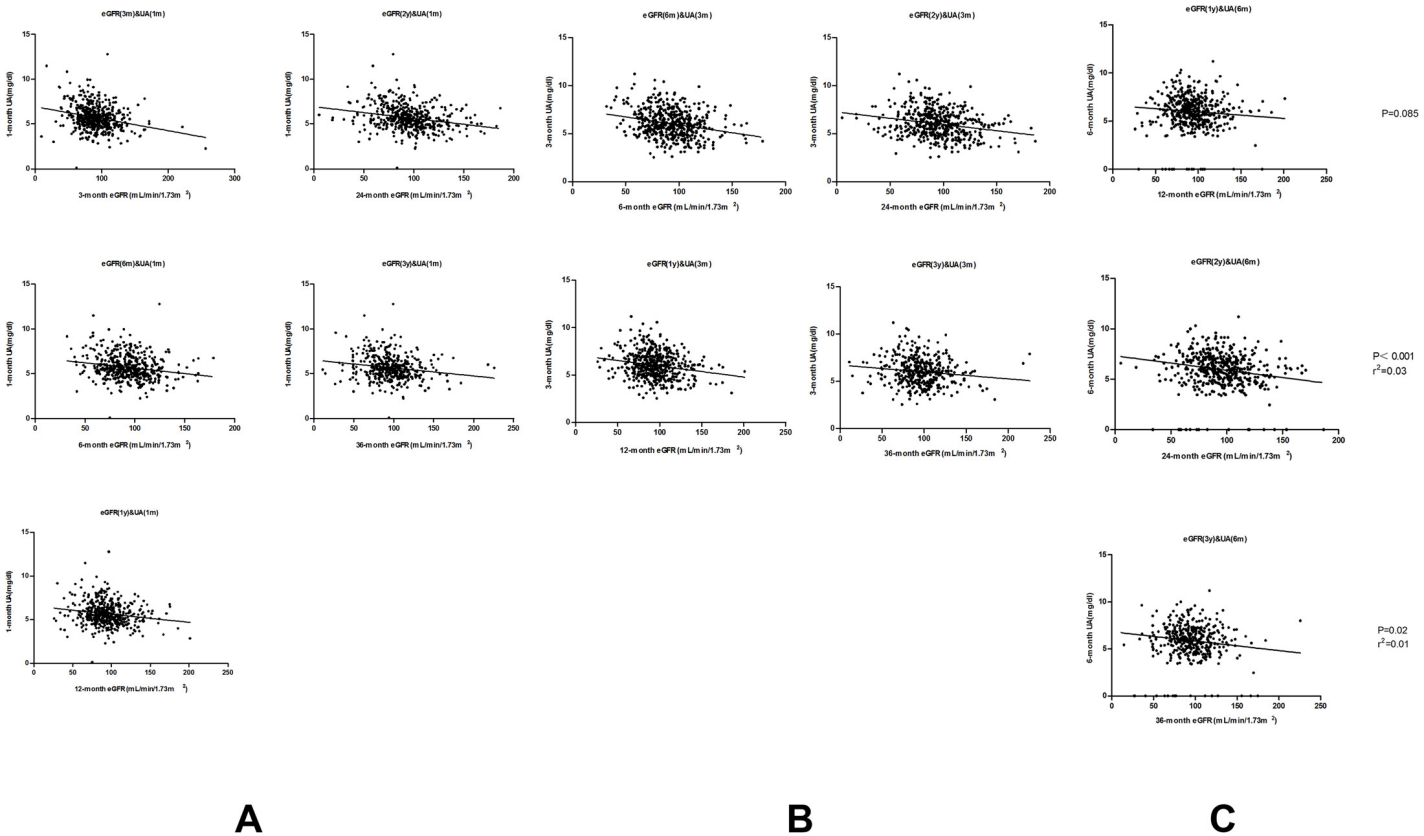
UA level predicts post-transplant kidney function. Scatter plots showed that UA level was negatively associated with eGFR at multiple times post-transplant. (A) This part showed the correlations between 1-month UA and 5 different time points eGFRs post-transplant. (B) The same correlation between 3-mont UA and 4 other time points. (C) 6-month UA cannot predict future eGFRs properly.

To examine the predictive value of UA level, we evaluated the mean eGFR levels calculated from SCr at multiple times post-transplant and UA levels obtained at 1-month, 3-month and 6-month respectively after surgery. As demonstrated in Fig 1A, 1B and 1C, 1-month and 3-month serum UA levels were associated with eGFR post-transplantseparately. All renal functions(demonstrated as eGFR) at different time post-transplant are negatively associated with early UA level, which indicates the predictive value of early UA level for renal function. Unfortunately, 6-month uric acid concentration did not correlates with 1-year eGFR anymore [see Fig 1C, P = 0.085]. Although this correlation with 2-year eGFR and 3-year eGFR was statistically significant, their degree of correlation was rather low (6-month uric acid with 2-year eGFR r^2^ = 0.03; 6-month uric acid with 3-year eGFR r^2^ = 0.01). Besides, 6-month post-transplant is getting away from the definition “early”. So we only recorded single-factor analysis between 1-month and 3-month UA level and eGFR. Considering eGFR would be affected by many factors including UA level, we further built up several regression equations to predict different time point eGFRs. The results are illustrated in Table 4. Each multiple regression was adjusted for UA, gender, BMI, age, introduction regimen, immunosuppressive agents, diabetic mellitus and triglyceride levels. Only significant factors after adjustment were included in Table 4. UA remained a predictor for eGFR in every regression equations.

**Table 4:** Prediction of renal function with early post-transplant UA levels using linear regression models. 95%CL, confidential interval for unstandardized B; r, correlation coefficient; Var, variables; B, unstandardized regression coefficient; β, standardized regression coefficient; Sex, presence for male gender and lack for female; multiple regression model includes UA, gender, BMI, age, introduction regimen, immunosuppressive agents, diabetic mellitus and triglyceride level; include criteria: P<0.05 and P>0.1 for exclude criteria;

	1-month								3-month							
	UA			Multiple regression					UA			Multiple regression				
	r	95% CL	P	Var	B	β	95% CL	P	r	95% CL	P	Var	B	β	95% CL	P
3-m eGFR	-0.25	-0.33 -0.16	***	UA	-4.4	-0.3	-5.88 -3.00	***								
				Sex	6.91	0.13	2.43 11.4	**								
				Age	-0.3	-0.1	-0.45 -0.63	**								
				BMI	-1.15	-0.15	-1.81 -0.49	**								
6-m eGFR	-0.19	-0.28 -0.11	***	UA	-2.98	-0.20	-4.28 -1.69	***	-0.25	-0.33 -0.17	***	UA	-3.4	-0.23	-4.68 -2.12	***
				BMI	-0.80	-0.12	-1.36 -0.24	**				BMI	-0.72	-0.11	-2.54 -0.01	*
12-m eGFR	-0.17	-0.25 -0.08	***	UA	-3.15	-0.17	-4.76 -1.55	***	-0.20	-0.28 -0.11	***	UA	-3.21	-0.18	-4.80 -1.62	***
				Sex	5.96	0.11	1.160.8	*				Sex	6.61	0.12	1.76 11.5	**
				BMI	-1.17	-0.15	-1.85 -0.49	**				BMI	-1.05	-0.14	-1.74 -0.36	**
24-m eGFR	-0.25	-0.33 -0.16	***	UA	-4.51	-0.24	-6.12 -2.91	***	-0.23	-0.32 -0.15	***	UA	-3.60	-0.20	-5.18 -2.01	***
				BMI	-1.00	-0.13	-1.69 -0.31	**				BMI	-0.91	-0.12	-1.61 -0.21	*
36-m eGFR	-0.17	-0.26 -0.08	***	UA	-3.41	-0.18	-5.19 -1.62	***	-0.14	-0.23 -0.04	**	UA	-2.71	-0.15	-4.52 -0.90	**
												Sex	5.82	0.10	0.30 11.3	*

***P<0.001

**P<0.01

*P<0.05

Acknowledging the predictive value of UA level, we then started to evaluate UA and its predictors. Serum UA was significantly relevant to age, male gender, DM comorbidity, cyclosporin use, RAS inhibitor use, diuretic use, TG level, rejection episode and, of course, eGFR. After adjustment for these variables, males with low eGFR but high level of TG who were on CSA, diuretics and RAS inhibitors and experienced at least one episode of acute rejection and diabetic issue were associated with a higher mean uric acid levels. When tested for odds ratio, low eGFR,medication prescription on CSA, diuretics and RAS inhibitors remained contributors to hyperuricemia (dichotomous form). More detailed results are displayed on Table 5.

**Table 5:** Factors could impact UA level and risk factors for hyperuricemia. Abbreviations as in Table 1; variables assignment for logistic regression: Dialysis type (prensence for peritoneal; lack for hemodialysis)

	Mean UA (mg/dL)				Hyperuricemia		
	B	β	95% CL	P	OR	95% CL	P
Age(y)	-0.588	-1.05	-0.99 to -0.18	0.005	1.02	0.99 to 1.04	0.144
Male	45.48	0.335	35.8 to 55.2	<0.001	0.77	0.46 to 1.29	0.324
BMI(kg/m^2^)	0.224	0.012	-1.24 to 1.69	0.765	0.96	0.88 to 1.03	0.242
DM	-21.56	-0.070	-42.9 to -0.24	0.047	0.41	0.11 to 1.48	0.174
Cyclosporin	11.06	0.087	2.17 to 19.95	0.015	1.64	1.01 to 2.67	0.046
Diuretics	44.20	0.257	32.4 to 55.9	<0.001	9.94	5.60 to 17.6	<0.001
RAS inhibitor	26.90	4.56	17.9 to 35.9	<0.001	3.08	1.92 to 4.92	<0.001
Follow-up time(m)	0.046	0.127	-0.20 to 0.29	0.719	1.00	0.99 to 1.02	0.242
Dialysis type	5.32	0.022	-11.5 to 22.1	0.534	2.14	0.99 to 4.61	0.053
DGF	0.253	0.001	-24.8 to 25.3	0.984	0.87	0.20 to 3.68	0.847
eGFR(mL/min/1.73m^2^)	-1.08	-0.359	-1.31 to -0.85	<0.001	1.04	1.02 to 1.05	<0.001
TG(mmol/L)	7.67	0.091	1.83 to 13.5	0.010	0.79	0.58 to 1.08	0.140
Rejection	-23.87	-0.104	-40.3 to -7.42	0.005	0.51	0.21 to 3.68	0.144
Infection	3.96	0.023	-8.04 to 15.9	0.518	1.69	0.92 to 3.10	0.09

### Clinical outcomes

**Fig 2:**
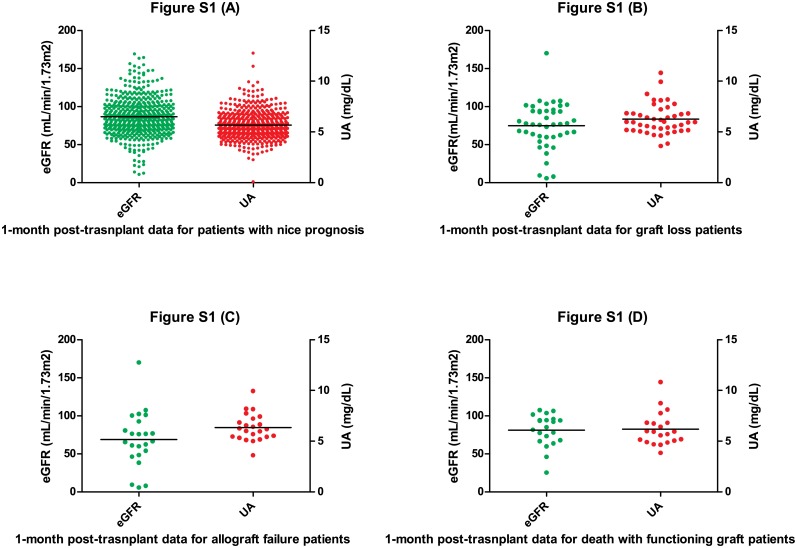
1-month post-transplant eGFRs and UAs for patients of different outcomes. Every single dot represents for either an eGFR or a UA value. Green dots are plotted on left y axis and red dots are on right y axis. (A) It indicates the group of patients without bad outcomes. (B) Patients suffered allograft failure or dead eventually. (C) Patients only suffered allograft failure. (D) Patients dead with functioning graft.

**Fig 3:**
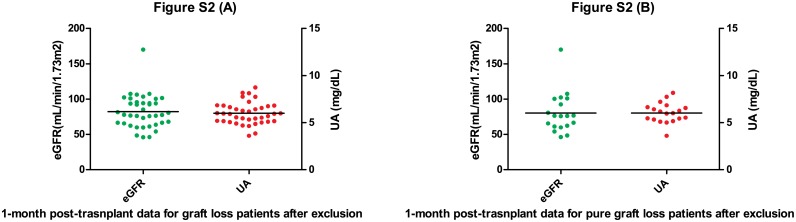
1-month post-transplant eGFRs and UAs after exclusion of 5 special cases. All eGFRs of the 5 patients are lower than 10. One of them was having an acute rejection when tested for eGFR, the other 4 patients were experiencing DGF, 2 of them returned to dialysis eventually and the other 2 had recovered 2 months later. (A) The group of recipients went through allograft failure or death eventually. (B) The group of patients only suffered allograft failure.

Early post-transplant outcome is also our concern when we initiated this proposition. Because the early post-transplant outcomes may determine the long-term outcomes. Therefore we need to distinguish different long-term prognosis groups as shown on Table 6 and Fig 2 to eliminate the bias caused by the early post-transplant outcomes. The graft loss patients have a 74.85 ± 30.44 eGFR level which is lower than patients without bad outcomes 86.65 ± 26.63 (P = 0.026). So does pure graft loss group when compared to normal recipients (69.0 ± 36.5 VS 86.65 ± 26.63, P = 0.006). These two groups have significantly lower mean eGFR mostly due to 5 special patients whose eGFR level were incredibly low (all eGFRs<10, 1 was having an acute rejection when tested for eGFR, the other 4 patients were experiencing DGF, 2 of them returned to dialysis eventually and the other 2 had recovered 2 months later). By excluding these 5 patients, mean eGFR and uric acid level of both groups are equally comparable with nice prognosis group (Table 7 and Fig 3).

**Table 6:** 1-month post-transplant eGFRs and UAs for patients of different outcomes. All P values are the results compared with recipients with nice prognosis group.

	Recipients with nice prognosis	Graft loss	P	Allograft failure	P	Death with functioning graft	P
1-month eGFR (mL/min/1.73m^2^)	86.65 ± 26.63	74.85 ± 30.44	0.026	69.0 ± 36.5	0.006	81.3 ± 21.0	0.708
1-month UA (mg/dL)	5.68 ± 1.35	6.26 ± 1.47	0.010	6.34 ± 1.35	0.013	6.18 ± 1.63	0.221

**Table 7:** 1-month post-transplant eGFRs and UAs after exclusion for 5 special cases. All P values are the results compared with recipients with nice prognosis group.

	Recipients with nice prognosis	Graft loss	P	Allograft failure	P
1-month eGFR (mL/min/1.73m^2^)	86.65 ± 26.63	82.23 ± 23.17	0.293	80.23 ± 28.58	0.129
1-month UA (mg/dL)	5.68 ± 1.35	5.99 ± 1.16	0.082	6.03 ± 1.06	0.102

**Fig 4:**
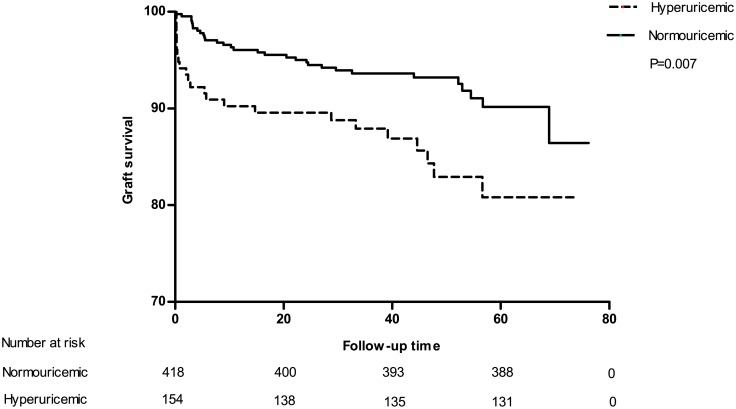
Kaplan-Meier survival curve estimates for graft loss. Hyperuricemic group survival curve was significantly (P = 0.007) lower than that of normouricemic group.Graft loss was defined as graft failure (return to dialysis) or death with functioning graft.

**Fig 5:**
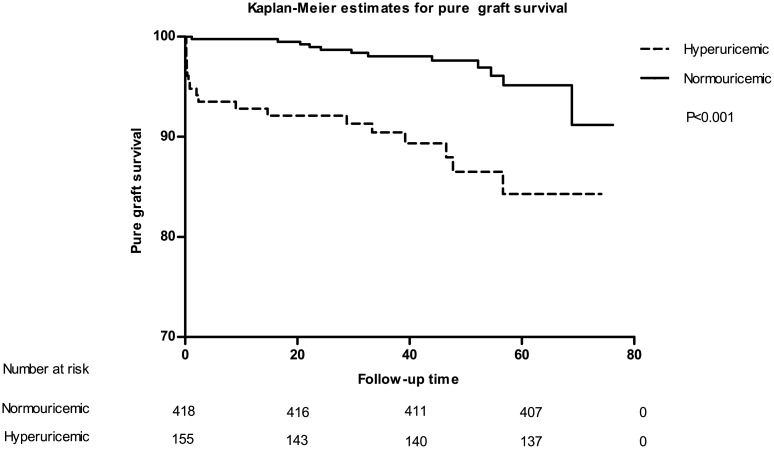
Kaplan-Meier survival curve estimates for pure graft survival. Excluding the dead with functioning kidney, we could observe greater variance between the two groups.

**Fig 6:**
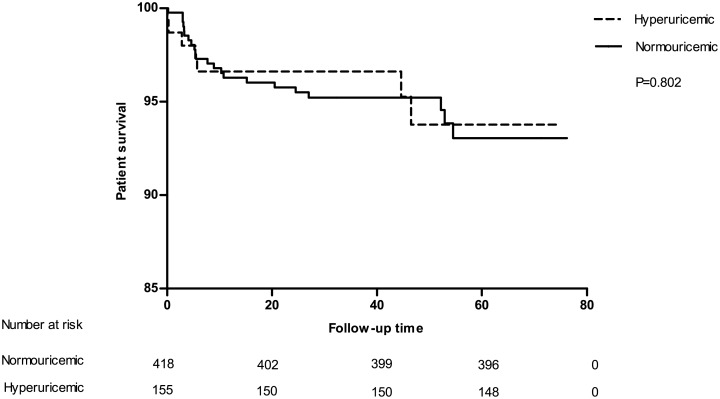
Kaplan-Meier survival curve estimates for death with functioning graft. No significant difference can be acquired on patient survival rate between these groups.

To estimate the impact of UA level and hyperuricemia on long-term transplant outcomes, Cox proportional hazard model was used to adjust for confounding factors. In accordance with our expectation, hyperuricemia was found to be a significant predictor for graft loss (defined as allograft failure and death) during the study period(hazard ratio [36] = 2.17, 95% confidential interval[CI]:1.27–3.70, P = 0.004) as illustrated in Fig 4. However, it was not valid(HR = 1.59, 95% CI: 0.73–3.44, P = 0.241) after adjustment for age, gender, BMI, HLA mismatch, introduction regimen, immunosuppressive agent protocol, diabetic mellitus, dialysis type, DGF, infection and acute rejection episode. Further investigation showed that hyperuricemia was significantly an independent predictor of pure graft failure (HR = 4.01, 95% CI: 1.25–12.91, P = 0.02) after adjustment for the same confounding factors as previous. Kaplan-Meier survival curve for allograft failure was depicted in Fig 5. Then we tested UA level as a continuous variable to double confirm our results. Similar analysis showed that UA level was also independently associated with allograft failure (HR = 1.009 for each unit increase in milligram per deciliter, 95% CI: 1.001–1.018, P = 0.026). When tested for graft loss, unlike hyperuricemia, UA level indicated borderline significant independently (HR = 1.005, 95% CI: 1.000–1.011, P = 0.052). Although significant, the HRs were too small to provide a strong evidence that uric acid, as a numeric variable, is associated to overall graft loss (allograft kidney failure and death with functioning graft) or pure graft loss. So we included all qualified recipients (the newly recruited consecutive cohort was made up of kidney transplantation recipients from 2005 to 2008) in our center, in total 1203, to figure out whether uric acid level is an independent risk factor in another Cox proportional hazard model. Due to low data integrity (the degree of low-integrity do not allow us to include these group of patients in other analysis, but it was good enough for us to perform the statistics described below), we had to narrow covariates down to age, gender, BMI, uric acid, DGF, infection and acute rejection episode. The results were encouraging. Uric acid level was independently associated with pure graft failure (HR = 1.121 for each unit increase in milligram per deciliter, 95% CI: 1.076–1.320, P = 0.015) after adjustment for covariates described above. But it was not significantly associated with graft loss (HR = 1.091, 95% CI: 0.898–1.210, P = 0.113) anymore. In contrast, death outcome did not show any correlation with both UA level and hyperuricemia which was obvious as demonstrated in Fig 6. And Table 8 provides all statistical evidences.

**Table 8:** Hazard ratios of graft loss, graft failure, and death with UA/hyperuricemia in Cox proportional hazard models. Graft loss includes allograft failure and death; multivariate variables include: age, gender, BMI, HLA mismatch, introduction regimen, immunosuppressive agent protocol, diabetic mellitus, dialysis type, DGF, infection and acute rejection episode; abbreviations as previous tables; HR, hazard ratio.

	Graft loss (n = 56)			Allograft failure (n = 30)			Death (n = 26)		
HR of outcomes for one mg/dL higher UA	HR	95% CL	P	HR	95% CL	P	HR	95% CL	P
Univariate	1.008	1.004–1.012	<0.001	1.013	1.007–1.018	<0.001	1.003	0.997–1.009	0.32
Multivariate adjusted	1.005	1.000–1.011	0.052	1.009	1.001–1.018	0.026	0.746	1.001–0.994	0.746
**HR of outcomes with Hyperuricemia**									
Univariate	2.17	1.273–3.699	0.004	4.192	2.018–8.705	<0.001	1.115	0.476–2.610	0.802
Multivariate adjusted	1.587	0.733–3.435	0.241	4.014	1.248–12.910	0.02	0.508	0.132–1.951	0.324

Unexpectedly, TG level (HR = 1.442for each unit increase millimoles per liter, 95% CI: 1.008–2.061, P = 0.045) was found to be an independent factor for graft loss. For pure graft failure outcome, covariates in Cox model with significance were rejection episode (HR = 25.828, 95% CI: 6.077–109.77, P<0.001), introduction use (HR = 3.491, 95% CI: 0.929–13.123, borderline significant P = 0.064) and hyperuricemia as mentioned previously. So were the results when UA was treated as a continuous variable (data not shown). Significant cause of death in both survival analysis with UA level and hyperuricemia, age (HR = 1.097, 95% CI: 1.039–1.158, P = 0.001), male gender (HR = 5.386, 95% CI: 1.101–26.336, P = 0.038), BMI (HR = 1.152, 95% CI: 0.982–1.352, borderline significant P = 0.082), infection episode (HR = 11.369, 95% CI: 4.049–31.921, P<0.001) and intriguing, meanwhile, unexpectedly TG level (HR = 1.717, 95% CI: 1.105–2.665, P = 0.016).

## Discussion

We observed that 3-month eGFR was statistically confined by UA level, gender, age and BMI collected at 1-monthpost-transplant. And the variables in the regression equations for medium-long renal function were different in number at different time points. One possible explanation: early stage of recovery from long-term dialysis and allograft compatibility issue could switch body metabolism pathway, therefore, more factors undergoing transform at early stage post-transplant might impact eGFR and more stabilized diet and lifestyle came along with less influencing factors. As previous reports [37, 38], obesity and metabolic syndrome are strongly associated with hyperuricemia likely as a consequence of insulin resistance, which explains larger BMI and higher TG level could elevate UA level. To our most curiosity, significant associations between hyperuricemia and overall/pure graft survival were observed, after adjustment for potential confounding variables. But the HRs were too small to infer UA was a risk factor. Even though our expanded investigation suggests that patients will have extra 1/10 chance to lead to allograft failure if their serum uric acid level increased 1 mg/dL, we still like to consider that post-transplant hyperuricemia is threatening the long-term outcome. Because hyperuricemia in this context means a long-time elevated serum uric acid exposure. Take all factors that contribute to overall graft loss into consideration, rejection episode (HR = 8.489, 95% CI: 3.502–20.578, P<0.001), infection episode (HR = 2.425, 95% CI: 1.175–5.008, P = 0.017) and DGF (HR = 3.228, 95% CI: 1.089–9.565, P = 0.035) played a dominant role, which could weaken the test power of hyperuricemia. So hyperuricemia may cause more troubles to patients without DGF, infection or rejection episode. So far, we have found that post-transplant hyperuricemia is threatening long-term graft survival and eGFR, CSA use, diuretic use and RAS inhibitor use could lead to hyperuricemia after renal transplantation. We may conclude that the medication we most usually prescribe to guarantee short-term outcomes of the recipients are compromising their long-term graft survival. An unexpected finding drew our attention that elevated TG level somehow declined the survival rate of allograft and patient’s mortality independently. Combining our findings, some old investigations[39, 40]and clinical experience, we guess that high TG level represents a risk factor for CV events which might be lethal, and plays a specific renal destructive effects [39]. This result provides a proof for aggressive management on hyperlipidemia after renal transplantation.

With controversial results, reports in the literature that focus on UA level on graft function/survival of renal transplantation are limited. Opposite to our observation, some investigators claim that UA level is generally irrelevant to renal function or allograft survival. Akgul et al. [41] did not find any differences in the development of CAN during first 3 years after transplantation between hyperuricemic and normouricemic recipients in a retrospective study of 133 patients with at least 6 months follow-up. Another retrospective study proposed by Meier-Kriesche et al. [42] in 2009 was a part of the Symphony study which enrolled 852 post-transplant patients. After corrected for baseline renal function, 1-month UA was not independently associated with 3-year renal function. The relationship between UA and the outcome was not performed. More recently, Numakura et al. [43] designed an observational study for Japanese population enrolled a sample size of 121 patients. The 1-yeareGFR was lower in patients with hyperuricemia, but graft survival did not differ between the patients with hyperuricemia treated with alloprinol and those without hyperuricemia.

On the contrary, Akalin et al. [44] investigated 307 renal allograft recipients for a mean 4.3 years of follow-up. They observed an association between hyperuricemia and several endpoint events including death, graft failure, new CV events, and biopsy-proven CAN during the follow-up time. UA level (HR: 1.12; p = 0.053) and hyperuricemia (HR: 1.69; p = 0.047) were associated with pooled outcome after adjusting for a number of variables including eGFR. Hyperuricemia was associated with the composite endpoint only in those with eGFR less than 50 ml/min/1.7m^2^. Haririan et al. [5] published their observational outcome in 2010 that UA level, as a continuous variable, and hyperuricemia, as a dichotomous variable, were associated with graft loss(HR: 1.26; p = 0.026 and HR: 1.92; p = 0.029, respectively) during 68 months (mean)follow-up in 212 living donor kidney transplant recipients. After a year, Haririan team demonstrated their further study on the same topic [45]. They enrolled 488 allograft recipients and traced for some time-varying variables for analysis. After adjustment for potential confounders that could affect the correlation results, UA was independently associated with increased risk of graft loss (HR: 1.15, p = 0.003; 95% CI: 1.05–1.27). In addition, UA and eGFR were detected an interaction relationship (HR: 0.996, p < 0.05; 95%CI: 0.993–0.999 for interaction term). A more comprehensive Meta-analysis proposed by Huang et al. [46]composites 12 cohort studies screened from 1417 articles by two reviewers found that renal transplant patients with hyperuricemia had lower eGFR (P<0.001, 95%CI:16.34,6.14) and higher SCr(P<0.001, 95%CI: 0.17,0.31) than those with normal uric acid level. And Meta-analysis showed that hyperuricemia was a risk factor of chronic allograft nephropathy (Unadjusted OR = 2.85, 95%CI: 1.84,4.38, adjusted HR = 1.65, 95%CI: 1.02,2.65) and graft loss (Unadjusted OR = 2.29, 95%CI: 1.55,3.39; adjusted HR = 2.01, 95%CI: 1.39,2.94).

The major advantage is that we did a relatively overall analysis with a reasonable sample size in the literature. Moreover, the hyperuricemia definition was designed for long-time exposure for transplanted kidney which is more convincing that the hazard factor influenced the recipient the whole time since operation. Additionally, we always compare UA, as a continuous variable and hyperuricemia, as a categorical variable with the outcomes of our interest to decline statistical bias. Another distinguishing aspect is that we introduced infection episode and rejection episode in our survival analysis. Though these two elements are known risk factors for poor outcomes, we still acquired the independent association between hyperuricemia and poor outcomes after adjusting these major risk factors, which intensifies our result. Interestingly, higher TG level, according to our results, is correlated with death and graft loss which contradicts the results of Gerhardt et al. [47].

The findings of our study should be interpreted with cautiousness. Because of its retrospective design, residual confounding cannot be excluded. Despite these limitations, this study has notable strengths and unique characteristics as detailed above.

In summary, we observed a significant association between serum UA level and poor outcomes after adjustment for confounders including infection and rejection episode. And early-stage post-transplant UA level can act as a predictor for renal function at multiple time points after transplant. Also, hypertriglyceride could lead to poor outcomes. Our findings bring up a question whether hyperuricemia management can be treated as a way to improve long-term prognosis of renal transplantation. And it may suggest that syndrome X leads to bad prognosis of renal transplantation. Further investigation are needed to examine if treatment for hyperuricemia, or maybe expand to syndrome X, could improve the outcome.

## Supporting Information

S1 File Original and English version blank sample of the *Red Cross Society of China Human Organ Donation Volunteers Registration Form*. The original form is provided by the Red Cross Society of China. The Red Cross Society coordinator will have a patient conversation with the potential donor’s immediate family about human organ donation with this file when unfortunate accident occurs. We translated it into English version.
(DOCX)

S2 File Original and English version pre-surgery consent form for living-related kidney donors. We made this consent form (Chinese and English version) to not only inform the living-related donor the risks he/she may confront but also rights and obligations of both sides of health care.
(DOCX)
