# Supplementary material for: Serum Uric Acid and Renal Transplantation Outcomes: At Least 3-Year Post-transplant Retrospective Multivariate Analysis
Source: PLoS One. 2015 Jul 24;10(7):e0133834. doi: 10.1371/journal.pone.0133834 (PMC4514650; doi:10.1371/journal.pone.0133834)
Supplement: S1 File — The original form is provided by the Red Cross Society of China. The Red Cross Society coordinator will have a patient conversation with the potential donor’s immediate family about human organ donation with this file when unfortunate accident occurs. We translated it into English version. (DOCX) [file pone.0133834.s001.docx]

| 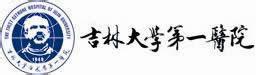 | | | | | | | | | | | | | | | | | | | | | | | | | | | | | |  |
| --- | --- | --- | --- | --- | --- | --- | --- | --- | --- | --- | --- | --- | --- | --- | --- | --- | --- | --- | --- | --- | --- | --- | --- | --- | --- | --- | --- | --- | --- | --- |
|  | | | | | | | | | | | | | | | | | | | | | | | | | | | | | |  |
| **亲属肾移植供者手术知情同意书** | | | | | | | | | | | | | | | | | | | | | | | | | | | | |  |  |
|  | | | | | | | | | | | | | | | | | | | | | | | | | | | | | |  |
| 患者姓名 | |  | | | 性别 |  | | | | | | 年龄 | | | |  | | | | | | | | | | | | | |  |
| 住院号 | |  | | | 病房号 |  | | | |  | | | 病床号 | | | | |  | | | | | | | | | | | |  |
| 术前诊断 | | | |  | | | | | | | | | | | | | | | | | | | | | | | | | |  |
| 拟行手术名称 | | | |  | | | | | | | | | | | | | | | | | | | | | | | | | |  |
|  | | | | | | | | | | | | | | | | | | | | | | | | | | | | | |  |
| **一般情况下捐献者在独肾状态下可维持正常生理机能及生活、工作，但面临的健康风险较双肾大，可能出现  肾功能受损，需进一步治疗，甚至血液透析治疗，且由于医学科学的特殊性和个体差异，在围手术期及远期可出现以下并发症，严重者可危及生命，如：** 1、各种感染（如伤口感染、肺部感染等）；2、麻醉意外；3、严重心律失常；4、手术中或术后出血；5、损伤邻近脏器（如气胸、肾上腺损伤、脾损伤、肠管损伤等）；6、血管栓塞（如股动脉栓塞、肺栓塞等）；7、切取的供肾热缺血时间过长、畸形、损伤，移植给受者后可能出现移植肾功能延迟恢复（DGF），急性排斥，以致移植物失功，严重者可能需切除移植肾；8、远期并发症如高血压、蛋白尿、切口疝、肠梗阻、慢性胰腺炎、肾炎、肾结石；9、气体栓塞等腹腔镜相关并发症  **除外以上手术意外及并发症，器官供受者及其家属应明确：** 1、接受移植者可以选择尸体供肾移植或其他肾脏替代治疗（如腹膜透析、血液透析等）；2、肾切除手术可能造成的手术风险不仅局限于健康本身，还包括供者受雇就业能力、保险以及无意识中对家庭和社会生活的影响；3、受者移植手术预后情况（良好的和不顺利的，详见同种异体肾移植手术同意书）和受者的特殊情况；4、捐赠者有保留自主考虑的权利，可以在捐赠过程的任何时间内否决已签定之志愿书，终止捐献，保证因为医学和自身因素而停止捐赠程序的原因将获得保密；5、肾脏捐赠完成后移植中心有义务监护外科手术后的整个恢复过程甚至供者情况稳定。 | | | | | | | | | | | | | | | | | | | | | | | | | | | | | |  |
|  | | | | | | |  | | 告知者（签字盖章）： | | | | | | | | | | | | | | |  | | | | | |  |
|  | | | | | | | | | | |  | | | | | | 年 | | |  | | 月 | | |  | | 日 |  | |  |
|  | | | | | | | | | | | | | | | | | | | | | | | | | | | | | |  |
| **患方意见：** | | | 告知清楚，同意手术。 | | | | | | | | | | | | | | | | | | | | | | | | | | |  |
| 手术中可能出现的危险、并发症等情况， 医师已向患者本人（ 患方）告知交待，患者方面充分考虑和理解可能遇到的手术风险，经权衡利弊，同意选择并接受该项诊疗服务。接受手术过程中，如遇不能独立行使知情同意权力的情况，同意由代理人行使知情同意权，具体委托见《患者知情同意权授权委托书》。 | | | | | | | | | | | | | | | | | | | | | | | | | | | | | |  |
|  | 患者签字或画押： | | | | | | |  | | | | | | 年 |  | | | | 月 | |  | | 日 | | |  | | | |  |
|  | 代理人签字或画押： | | | | | | |  | | | | | | 年 |  | | | | 月 | |  | | 日 | | |  | | | |  |
|  | | | | | | | | | | | | | | | | | | | | | | | | | | | | | |  |

吉大医表字[2002]012号

| 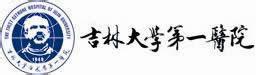 | | | | | | | | | | | | | | | | | | | | | | | | | | | |  |
| --- | --- | --- | --- | --- | --- | --- | --- | --- | --- | --- | --- | --- | --- | --- | --- | --- | --- | --- | --- | --- | --- | --- | --- | --- | --- | --- | --- | --- |
|  | | | | | | | | | | | | | | | | | | | | | | | | | | | |  |
| 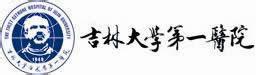 | | | | | | | | | | | | | | | | | | | | | | | | | | | |  |
|  | | | | | | | | | | | | | | | | | | | | | | | | | | | |  |
| **Living-related kidney donor pre-surgery consent form** | | | | | | | | | | | | | | | | | | | | | | | | | | |  |  |
|  | | | | | | | | | | | | | | | | | | | | | | | | | | | |  |
| Full name | |  | | | Gender |  | | | | | Age | | |  | | | | | | | | | | | | | |  |
| Inpatient No | |  | | | Room |  | | | | | Bed | | | | |  | | | | | | | | | | | |  |
| Pre-surgery diagnosis | | |  | | | | | | | | | | | | | | | | | | | | | | | | |  |
| Operation name | | |  | | | | | | | | | | | | | | | | | | | | | | | | |  |
| **Normally, solitary kidney status could maintain donor’s physical function, however, living donor may suffer from more health risks compared to both-kidney people. Kidney function** **deterioration may occur, and further renal replacement therapy (for example: dialysis) might be followed. Because of medical science’s feature and individual difference, donor may suffer from the following complications during** **perioperative and long-term period:**  1. Infections (for example, incision infection, pulmonary infection);  2. Anesthetic accident;  3. Severe arrhythmia;  4. Bleeding during/post surgery;  5. Adjacent organs injury (for example, pneumothorax, adrenal gland injury, spleen injury, intestines injury and so on);  6. Thrombosis (for example, femoral artery embolism, pulmonary embolism).  7. Because of long cold ischemic time, deformity or injuries, the recipients may have Delayed Graft Function (DGF), acute rejection. These issues may cause graft failure and the graft kidney might be removed;  8. Long-term complications like hypertension, proteinuria, incisional hernia, intestinal obstruction, chronic pancreatitis, nephritis, renal calculus);  9. laparoscope related complications like air embolism.  **Except for surgical accidents/complications mentioned above, kidney donor and his/her family should also be noted:**  1. Graft recipients can certainly choose to receive DCD donor’s kidney or other renal replacement therapy (for example, hemodialysis or peritoneal dialysis);  2. Nephrectomy may cause not only surgical risks for donors but also employability, health insurance and unconscious effect for his/her family and the society;  3. Graft recipient’s outcome and other exceptional cases can be found on the *Living-related kidney recipient’s pre-surgery consent form*;  4. Kidney donors have the right to retain independent consideration which totally allows them to revoke the already-signed volunteer’s application form at any moment before the surgery carries out, so that the donation process will be ended. We guarantee that the reason that he/she wants to cancel the donation process will be kept confidential if the reason is due to medical or personal factors;  5. It is our transplant center’s obligation that we monitor the donor’s recovery till he/she fully healed. | | | | | | | | | | | | | | | | | | | | | | | | | | | |  |
|  | | | | | | |  | | announcer （sign and seal）： | | | | | | | | | | | | | | | |  | | |  |
|  | | | | | | | | | |  | | | | |  | | | Date: | |  | |  | |  | |  | |  |
|  | | | | | | | | | | | | | | | | | | | | | | | | | | | |  |
| **Patient/Family’s opinion：** | | | |  | | | | | | | | | | | | | | | | | | | | | | | |  |
| Dr._____ has fully informed the donor/agent party the possibility of surgery related complications and risks. I, now, adequately understand the relevant danger after weighting the advantage and disadvantage. I choose to agree and accept this particular medical service. If there is a chance that I may not exercise my informed consent right independently, my assigned agent would do it for me. See more detailed stipulations on the *Letter of authorization* | | | | | | | | | | | | | | | | | | | | | | | | | | | |  |
|  | Patient (signature)： | | | | | | | Date | | | |  |  | | | |  | |  | |  | |  | | | | |  |
|  | Agent (signature)： | | | | | | | Date | | | | ==== |  | | | |  | |  | |  | |  | | | | |  |
|  | | | | | | | | | | | | | | | | | | | | | | | | | | | |  |

[2002]No 012
