# Supplementary material for: Serum Uric Acid and Renal Transplantation Outcomes: At Least 3-Year Post-transplant Retrospective Multivariate Analysis
Source: PLoS One. 2015 Jul 24;10(7):e0133834. doi: 10.1371/journal.pone.0133834 (PMC4514650; doi:10.1371/journal.pone.0133834)
Supplement: S2 File — We made this consent form (Chinese and English version) to not only inform the living-related donor the risks he/she may confront but also rights and obligations of both sides of health care. (DOCX) [file pone.0133834.s002.docx]

| 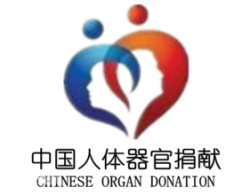 |
| --- |
| 人体器官（遗体、组织）捐献登记表填写须知 |
|  |
| 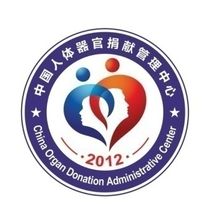**中国人体器官管理中心** 制作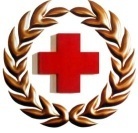**中国红十字会** 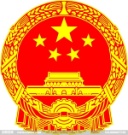**中华人民共和国国家卫生和计划生育委员会**  感谢你们在家庭承受着巨大痛苦的时候，愿意代表你们的家人做出器官捐献的决定。这将挽救很多人的生命，给许许多多的家庭带来希望。请仔细阅读以下内容：  1、器官捐献就是当一个人不幸去世时，根据本人或亲属意愿，将其功能良好的器官，以无偿的方式，捐献给器官功能衰竭的患者，让他们能够延续生命，改善未来生活质量，并能继续贡献社会。中国红十字会开展的人体器官捐献试点工作是指面向社会公众在其身故后自愿的器官捐献，不涉及活体器官捐献。  2、器官捐献并无绝对的年龄限制，要求志愿捐献者没有感染艾滋病或其他严重传染病，没有癌症（原发脑肿瘤除外），具体由人体器官捐献专家组、人体器官评估组织评估器官、组织是否适合捐献。  3、根据《人体器官移植条例》（国务院令491号）规定，人体器官捐献必须遵循自愿无偿的原则。包括两种情况：①有完全民事行为能力的公民通过书面自愿申请器官捐献登记，而且并没有撤销该登记，待其身故后进行器官捐献；②公民生前未表示不同意捐献器官，待其身故后，其配偶、成年子女、父母以书面的形式共同表示愿意捐献器官。  4、亲属有权决定是否捐献器官及捐献何种器官，有权在捐献手术之前以书面的形式撤销、更改捐献登记。  5、器官捐献会严格地按照法律程序和医疗程序进行，任何时候都不会影响志愿捐献者的抢救和治疗。器官摘取时采用严格的外科手术标准，如同在医院接受任何一项外科手术。在整个过程中，医护人员绝对尊重捐献者的遗体、遗容，并最后回复遗体原貌。  6、凡捐献者去世后因器官捐献而产生的费用，均无须亲属承担。  7、基于对捐献者和接受者的隐私权，捐献者和接受者双方的信息都将严格保密，以避免当事人产生不必要的困扰。如果双方同意，相关的工作人员会告知捐献者家人有关器官接受者手术后的进展情况。  8、亲属在填写登记表时需携带有效身份证件及与捐献者的关系证明，共同在登记表上签字。  9、《人体器官（遗体、组织）捐献登记表》一式两份，分别由吉林省人体器官捐献办公室和捐献者家属保存。 |
|  |
|  |

|  |
| --- |

| 中国红十字会人体器官捐献志愿者申请登记表 | | | | | | | | |
| --- | --- | --- | --- | --- | --- | --- | --- | --- |
| 姓名 |  | | 性别 | （男/女） | | 出生年月 | |  |
| 血型 | （A/B/O/AB/RH+/RH－） | | | | | 身份证号码 | |  |
| 学历 |  | | | | | 职 业 | |  |
| 志愿捐献器官（组织）：眼角膜（ ） 心脏（ ） 肺脏（ ） 肝脏（ ） 肾脏（ ） 胰腺（ )其它（ ）（用√选择，可同时选多项） | | | | | | | | |
| 是否捐献遗体 | | 是（ ） 否（ ） | | | | 登记地点 | |  |
| 联系电话 | |  | | | | 直系亲属姓名 | |  |
| 地址 | |  | | | | 亲属联系电话 | |  |
| 邮 编 | |  | | | | 直系亲属是否知情：是（ ） 否（ ） | | |
| E-mail | |  | | | | | | |
| 其他 | | 以上资料是否保密 | | | 是（ ） | | 否（ ） | |
|  |  | 是否愿意配合宣传 | | | 愿意（ ） | | 不愿意（ ） | |
|  |  | 是否需要宣传资料 | | | 需要（ ） | | 不需要（ ） | |
|  |  |  | | |  | |  | |
| 本人郑重承诺：我志愿成为中国红十字会器官捐献志愿者，在身故后愿意无偿捐献以上所选器官（组织），用于需要移植的患者或医学事业，请尊重我的决定。  **志愿者签名： 年 月 日** | | | | | | | | |

编号：


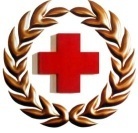


声明：填写此表格仅仅是为了完成您自愿捐献人体器官的意愿。您可以随时通过书面形式撤回已做出的捐献意愿。没有您的同意，我们不会将您的个人信息传播给第三方，除非我们依法必须交出。

| 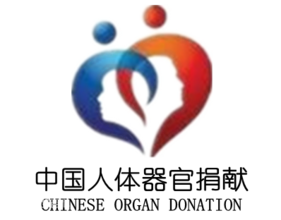 |
| --- |
| Notes for Human Organ (remains, tissue) Donation |
|  |
| 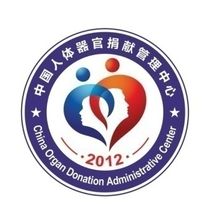**中国人体器官管理中心** 制作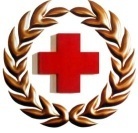**中国红十字会** 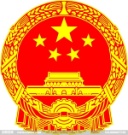**中华人民共和国国家卫生和计划生育委员会**  Thank you for willing to make this decision of human organ donation on behalf of your family at the time that you are under such a tremendous sorrow. Your kind benefaction is going to help a lot of souls and bring hopes for so many families. Please, read the following content carefully:  1、Organ donation is when someone unfortunately departures the world, depending on his/her own or family’s will, donating his/her well functioning organ to another organ failure patient, so that the recipient may extend their lifespan, develop life quality and continue to contribute to the society. The Red Cross Society of China Human Organ Donation Project is oriented to citizen who wants to donate their organ(s) after their death. We DO NOT involve living organ donation issues.  2、Donation criteria do not include absolute age limit. However we have to make sure that the donor should NOT have AIDS or other severe infectious disease, cancers (exclude primary brain tumors). Specific qualification criteria will be made by human organ donation expert group and human organ assessment group.  3、According to *regulations on human organ transplantation* (the state council order no. 491), human organ donation must comply with the voluntary unpaid principle. Two cases may occur: ①Citizens with full capacity for civil conduct may become an organ donor after their death by applying the *Human Organ Donation Volunteers Registration Form* and without revoking; ②Citizens had not declare unwilling to organ donation during his/her lifetime may donate their organ(s) by the common consent of his/her spouse, adult children and parents in written form after death.  4、Relatives of potential donors have the right to decide whether to proceed organ donation or not, what kind of organ to donate, and the right to revoke or alter the donation registration in written form.  5、Organ donation will strictly follow legal and medical processes. Any action in any form that interferes the rescue and treatment will not be tolerated. The procedure of organ harvest will sternly stick to surgical standards as much as any other surgical managements. During the whole process, medical care personnel will pay absolute respect to the donor’s remains. Body appearance will be restored in the end of organ harvest.  6、Relatives do not need to bear the cost that comes from the post-donor’s death issues.  7、Considering donor’s and recipient’s privacy, their information will be kept strictly confidential so both parties could avoid unnecessary harassment. If a mutual agreement is reached, relevant staff can totally update the recipient’s information to the donor’s family.  8、Relatives of donors will bring their valid identifications and certificates of kindred when filling in the registration form and cosign it, should the form become effective.  9、*Human Organ (remains, tissue) Donation Volunteers Registration Form* will be done in duplicate. And they will be saved by the Human Organ Donation Office of Jilin Province and the donor’s family. |

No：


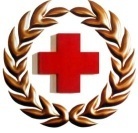


| **Red Cross Society of China Human Organ Donation Volunteers Registration Form** | | | | | | | | | |
| --- | --- | --- | --- | --- | --- | --- | --- | --- | --- |
|  |  |  |  |  |  |  |  |  |  |
| Name |  | Gender | （male/female） | Date of birth | | |  | | |
| Blood type | （A/B/O/AB/RH+/RH－） | | | ID number | | |  | | |
| Education |  | | | Occupation | | |  | | |
| Organ willing to donate（tissue）：cornea（ ） heart（ ） lung（ ） liver（ ） kidney（ ） pancreas（ )others( ）（pick with√） | | | | | | | | | |
| Donate remains | | | Yes（ ） No（ ） | | Registration location | | | |  |
| Contact number | | |  | | Name of next of kin | | | |  |
| Address | | |  | | Family contact number | | | |  |
|  |  |  |  |  |  |  |  |  |  |
| Post code | | |  | | Is next of kin informed: Yes（ ） No（ ） | | | | |
| E-mail | | |  | | | | | | |
| Others | | | Confidential need | | | Yes（ ） | | No（ ） | |
|  |  |  | Willing to advertise | | | Yes（ ） | | No（ ） | |
|  |  |  | Need advertise materials | | | Yes（ ） | | No（ ） | |
|  |  |  |  | | |  | |  | |
| I solemnly declare that I volunteer to become an Organ Donor of the Red Cross Society of China. Please, respect my decision that I am going to donate the organ (tissue) picked above for patients in need or other medical purposes after my death. | | | | | | | | | |
|  | | | | | | | | | |
| **Volunteer’s signature： Date** | | | | | | | | | |

Declaration：Filling in this form only means that you volunteer to donate your organ. You may revoke your will in written form at any moment. We will not spread your personal information to third party unless legal forced us to.
